# Draft Genome Sequence of CH_48, a Highly Cytotoxic Bacillus thuringiensis Strain Isolated from Rosemary at the Retail Level

**DOI:** 10.1128/MRA.00751-20

**Published:** 2020-08-06

**Authors:** Marc J. A. Stevens, Sophia Johler

**Affiliations:** a Institute for Food Safety and Hygiene, Vetsuisse Faculty, University of Zurich, Zurich, Switzerland; Queens College

## Abstract

Bacillus thuringiensis CH_48 exhibits extremely high levels of Vero cell cytotoxicity and sphingomyelinase activity.

## ANNOUNCEMENT

Bacillus cereus *sensu stricto* and Bacillus thuringiensis are genetically intermingled, with B. thuringiensis exhibiting additional toxin genes that encode crystal toxins with insecticidal properties (1, 2). While Bacillus cereus *sensu stricto* is recognized as a major cause of gastroenteritis, the role of the closely related B. thuringiensis in foodborne disease remains a topic of controversy. Still, B. thuringiensis strains are widely used as biopesticides.

In this study, we present the complete genome sequence of B. thuringiensis CH_48. This strain was isolated in a study that investigated the characteristics of B. thuringiensis isolates from biopesticides, food, and foodborne outbreaks (3). CH_48 was isolated from fresh rosemary that was purchased at a Swiss supermarket in 2016 (3). The strain exhibited cytotoxic effects 1.5× higher than those of the highly toxic B. cereus sensu stricto reference strain NVH 0075-95 and in addition produced very high levels of sphingomyelinase (3).

Chromosomal DNA was extracted from an overnight culture of a single colony on brain heart infusion (BHI) agar at 30°C using a DNA blood and tissue kit (Qiagen, Hombrechtikon, Switzerland). A transposome-based library was prepared for Illumina MiniSeq paired-end sequencing using the Nextera DNA Flex sample preparation kit (Illumina, San Diego, CA, USA). Two data sets were obtained, each containing 1,446,327 reads of 150 bp, corresponding to an estimated coverage of 110-fold. The reads were assembled using Shovill version 1.0.9, a software tool that uses SPAdes for genome assembly (4, 5). Standard settings were used, and the minimal contig size was set at 1,000 bp. The genome of CH_48 consists of 65 contigs with a total length of 5,834,184 bp. The *L*_50_ value was 5, and the *N*_50_ value was 451,251 bp; the largest contig was 872,014 bp. The GC content is 38.8%. The genome was annotated with PGAP by NCBI. The genome of CH_48 comprises 5,930 genes, with 5,649 protein coding sequences and 93 tRNAs.

### Data availability.

The sequence and annotation data of B. thuringiensis strain CH_48 were deposited in GenBank under the accession number JABERC000000000. Reads were deposited to the Sequence Read Archive under the BioProject accession number PRJNA629343.
